# Reduction of early surgical site and other care related infections in 3553 hip fracture patients: lessons learned from the 5-year Safe Hands project

**DOI:** 10.1186/s13756-022-01153-4

**Published:** 2022-09-05

**Authors:** Annette Erichsen Andersson, Brigid M. Gillespie, Magnus Karlsson, Henrik Malchau, Bengt Nellgård, Ewa Wikström, Cecilia Rogmark, Jonatan Tillander

**Affiliations:** 1 Institute of Health Care Sciences, Sahlgrenska Academy, University of Gothenburg, Box 457, 405 30 Gothenburg, Sweden; 2 Department of Orthopedics, Sahlgrenska University Hospital, Gothenburg, Sweden; 3 NHMRC Centre of Research Excellence in Wiser Wounds, Menzies Health Institute, Queensland, Griffith University & Gold Coast University Hospital and Health Service, Queensland, Australia; 4 Department of Anesthesiology and Intensive Care Medicine, Sahlgrenska University Hospital, Gothenburg, Sweden; 5 Department of Business Administration, School of Business, Economics and Law, University of Gothenburg, Gothenburg, Sweden; 6 Department of Orthopedics, Skane University Hospital, Lund University, Malmö, Sweden; 7 Swedish Hip Arthroplasty Register, Registercentrum VGR, Gothenburg, Sweden; 8 Department of Infectious Diseases, Sahlgrenska University Hospital, Gothenburg, Sweden

**Keywords:** Surgical site infections, Hip fracture, Hip surgery, Infection prevention, Implementation research, Knowledge translation

## Abstract

**Background:**

Surgical site infection (SSI) after acute hip fracture surgery is a devastating complication associated with increased suffering and mortality. The aim of the study was to investigate early SSI, sepsis, pneumonia and urinary tract infections over five years, before and after the implementation of the *Safe Hands* project.

**Methods:**

This was a single-centre observational study with a 5-year longitudinal design, investigating the effects of an infection-prevention intervention targeting the clinical care pathway of individuals with acute hip fracture. Statistical analyses were based on routinely collected patient outcome data comprising 3553 patients. The study conforms to the criteria of the Strengthening the Reporting of Observational Studies in Epidemiology (STROBE).

**Results:**

The incidence of early SSIs decreased from 2.5% in years 1–2 to 1.1% in years 4–5. Similar results were observed for sepsis (2.7% to 1.3%) and urinary tract infections (14.2% to 4.2%). The multivariable regression results suggest that, for every observed year, the odds of early SSIs decreased. Male gender, procedure time, sepsis and preoperative skin damage increased the odds significantly.

**Conclusions:**

Our preventive bundle, based on partnership between researchers, managers and clinicians and a strong commitment to change from the involved professions, appear to be effective in reducing the frequency of potentially devastating SSIs and other hospital acquired infections after hip fracture surgery. The use of external and internal facilitators was crucial to enable individual and organisational learning and overcoming barriers to improvements.

*Trial registration:* Clinical Trials.gov ID: NCT02983136 Registered 6 December 2016—Retrospectively registered.

## Background

Surgical site infection (SSI) after acute hip fracture surgery is a devastating complication associated with increased suffering and mortality [1, 2]. The reported infection rates vary between 1.7% and 10% in relation to diagnostic criteria, fixation method and follow-up [3–5]. Suffering a hip fracture is associated with an increased risk of death in the first year after surgery [6, 7] The consequences in terms of hospital costs and resources are substantial in terms of prolonged hospital length of stay (HLOS), re-operations and extra medication [8]. Patients with hip fractures are generally older, frail and have multiple co-morbidities such as chronic obstructive pulmonary disease, diabetes, and dementia. The abovementioned conditions are established patient-related risk factors for SSI, but most are inherently less modifiable than in elective procedures [3, 6, 9, 10].

Modifiable independent risk factors such as operating time are well described, while the use of drainage is unsettled [5]. In previous studies, we have found unjustified differences in intra-operative care between different surgical methods. Preventive measures were not used to the same extent in hip fracture surgery compared with primary hip or knee arthroplasty [11]. A general lack of hand hygiene and aseptic techniques in the operating room (OR), especially during anaesthetic care, was identified [12, 13], together with the fact that organisational structures, conflicting goals, and hierarchical issues often worked as barriers to change [14]. In cases of success, supportive relationships between the managers from different professions and organisational levels were essential, along with a strong sense of ownership and control over the implementation process [14]. Given the prevalence of hip fractures, patients’ vulnerability, and their need for surgery to regain functional independence, there was a strong commitment among hospital management and researchers to improve the quality of care. To address the identified contextual problems, the *Safe Hands* project was initiated (ClinicalTrials.gov ID: NCT02983136), This project aimed to test and evaluate an implementation programme to increase awareness of hospital-acquired infections (HAI) and increase the use of preventive measures including hand hygiene, with the emphasis on the vulnerability of patients with hip fractures. The programme had an iterative, flexible approach, allowing for the co-creation of solutions and adaptations to the specific contextual factors. The implementation process for components in the programme and the links to theory have previously been published [15, 16]. In short, we used a theory driven approach to motivate and engage managers, leaders, and health professionals in the project. Schein’s theories on organizational culture, leadership, and change [17] and Isaacs work on dialogue [18] was used as a foundation for the development of the integrated knowledge translation (iKT) program. The core of iKT is partnership between the researchers, health professionals and stakeholder. Thus, the participants were involved early in the planning of the project and collaborated throughout the project. The use of facilitators [19, 20] was crucial to help overcome contextual and cultural barriers and enable managers and health care professionals to participate in the project. These partnerships also facilitated interprofessional dialogues to create shared goals around infection prevention.

The *Safe Hands* project was expanded to involve the entire hip fracture care pathway, targeting catheter-related urinary tract infections (UTI), the risk of bladder distention and deficits in hand hygiene and aseptic techniques. The aim of this study was to investigate early SSI, sepsis, pneumonia, and urinary tract infections over five years, before and after the implementation of the *Safe Hands* project.

## Methods

### Design and setting

This single-centre observational study used a five-year longitudinal design. The study was conducted at the largest orthopaedic university hospital in Scandinavia. Statistical analyses were based on routinely collected patient outcome data. This study conforms to the criteria of the Strengthening the Reporting of Observational Studies in Epidemiology (STROBE) [21].

No alteration in antibiotic prophylaxis was introduced across the study period. At our center a single dose of cloxacillin for internal fixation and 3 doses for arthroplasties is standard. The blood glucose protocol states that the goal is to keep the levels between 5–8 mmol/L, but > 4 and < 12 mmol/L are accepted. Before draping, the incision site was prepped with an alcoholic chlorhexidine solution (5 mg/ml). Double gloves were used, and the outer gloves were changed if indicated. Fixation with gentamicin-loaded bone cement was used in all hemiarthroplasties. All instrument nurses were registered nurses (RNs) with a specialisation in perioperative care (one-year master’s degree).

### Data source

All patients recorded in the hospital’s quality registry from year 1 (May 2015) to year 5 (March 2020) were eligible for inclusion, (Year 1: baseline, year 2–3: intervention phase and year: 4–5 follow up). After March 2020, Covid-19 hit the hospital with full force, consequently not eligible for inclusion in the present study. The inclusion criteria were: (i) age > 65 years, (ii) surgically treated hip fracture and (iii) perioperative care at an orthogeriatric ward in our hospital. Exclusion criteria: (i) HLOS < 2 days, (ii) pathological fracture, (iii) excision arthroplasty and (iv) re-fracture or a contralateral hip fracture. Variables and definitions are presented in Table 1. The hospital’s quality register contains prospectively collected in-hospital data related to orthogeriatric hip fractures.

**Table 1:** Variables and definitions

Age	Years
Gender	Female/male
Surgical method	Total arthroplasty Hemiarthroplasty Internal fixation with osteosynthesis
American Society of Anaesthesiologists’ (ASA) classification of physical health	I-IV ASA I-no systemic disease ASA II-mild systemic disease ASA III-moderate systemic disease ASA IV-severe systemic disease that is a constant threat to life
Early surgical site infection	a) Development of a superficial or deep infection in the surgical wound any time up to hospital discharge b) Diagnosed by a physician and treated with antibiotics with or without surgical revision
Urinary tract infection, pneumonia and sepsis	Data were extracted from diagnosis codes at discharge from the hospital and based on physician diagnosis and treated with antibiotics Infections were deemed as hospital related if they occurred > 2 days after admission to hospital
Cognitive failure	Including temporary impairment and dementia
Diabetes	Type II: any type of treatment
Pressure ulcer	Norton stage I-IV
Intact skin	Recorded on admission
Time to surgery	Measured from in-hospital diagnosis to surgery dichotomised: < or > 36 h
Housing before hospitalisation	Home or nursing home

### The bundle intervention and changes in standard practices

Ongoing interventions in complex hospitals settings can yield both positive synergies and competing interests and conflicting goals within the organisation. This paper considers this complexity by reporting all the relevant method and organisational changes occurring during the five-year study period. For example, two to three preoperative showers with 4% chlorhexidine gluconate have been part of the hospital preoperative protocol for patients undergoing orthopedic implant surgery for > 30 years like in most Swedish hospitals. The changes from two to one was not a part of our intervention but we choose to take this change into account as it might influence the outcome. We expanded the Safe Hands project in close cooperation with the stakeholders, managers, and health professionals to prevent catheter-related urinary tract infection in an intervention called Safe Bladder consisting of a bundle of preventive measures [22]. The components of the *Safe Hands* project and other changes in hospital standard practices are presented in Table 2.

**Table 2:** Timeline of the *Safe Hands* project (italic) and changes in standard practices

Year and quarters	Interventions and changes in routines
1 Baseline Q1-2	*Systematic collection of outcome data and related variable after hip fracture surgery*
Q 3–4	Introduction of the *Safe Hands* project (ClinicalTrials.gov ID: NCT02983136) to secure leadership commitment to infection prevention in surgery
	A new routine promoting early assessment by the consulting infectious diseases specialist in *Staphylococcus aureus* bacteraemia was introduced
2 Intervention Q1-2	*The Safe Hands project was launched in the OR*
Q3-4	*A new routine was implemented that formalised the practice that junior physicians in training should receive support from a senior surgeon to avoid prolonged surgical time for hip fracture surgery. The aim was to create a culture where it would be easy and appropriate to ask for help from a senior*
3 Intervention Q1-2	Antibiotic rounds twice weekly led by a consulting infectious diseases specialist were introduced on the geriatric wards with the aim of promoting sound antibiotic use, e.g. reducing the number of prophylaxis-resistant bacterial strains on the wards Accessibility to the consulting infectious diseases specialist was increased from two to four days a week for bedside assessments The preoperative shower routine consisting of a double shower with 4% chlorhexidine gluconate (CHX) was changed from two showers before surgery to one shower before surgery^a^ If the patient had to wait for surgery for more than 48 h after the first shower, an additional CHX treatment was carried out
	*Expanding the Safe Hands project; a catheter-related urinary tract infection prevention strategy (Safe Bladder) was developed*
Q3-4	*Safe Bladder was implemented in the full care pathway ER, OR, PACU and the geriatric wards*
4. Post-intervention Q1-2	
Q 3–4	
5 Post-intervention Q1-2	The antibiotic rounds led by a consulting infectious diseases specialist were reduced from twice to once weekly
Q3-4	

### Data collection

Prospectively collected register data over five years were used to analyse early SSIs and other infectious outcomes. In addition, other organisational changes unrelated to the *Safe Hands* project were recorded. The data files were scrutinised by a research nurse and missing data were corrected in the register whenever possible. Outliers were verified against source data (i.e. patient records). The data files were cleaned according to the inclusion and exclusion criteria, outlined in Table 3 for included and excluded patients.

**Table 3:** Included and excluded subjects with reasons in years 1–5

Period	Year 1 Baseline	Year 2 Intervention	Year 3 Intervention	Year 4 Follow-up	Year 5 Follow-up
Eligible	n = 461	n = 833	n = 842	n = 775	n = 741
Total excluded	n = 16	n = 23	n = 21	n = 5	n = 3
Reasons for exclusion	Surgery at another hospital, n = 8 Wrong fracture, n = 5 Girdlestone, n = 3	Wrong fracture, n = 4 Girdlestone, n = 3 Registered twice, n = 7 < 65 years of age, n = 3 < 2 HLOS^a^, n = 3	Surgery at another hospital, n = 3 Wrong fracture, n = 5 Girdlestone, n = 6 < 65 years of age, n = 2 < 2 HLOS^a^, n = 5	Wrong fracture, n = 2 < 65 years of age, n = 2 < 2 HLOS^a^, n = 1	Wrong fracture n = 1 < 65 years of age = 1 < 2 HLOS^a^ = 1
*Included*	*n* = *442*	*n* = *820*	*n* = *806*	*N* = *733*	*n* = *712*
Total sample	N = 3553				

### Statistical methods

Primary outcome: early SSI, secondary outcomes: sepsis, pneumonia and urinary tract infection(UTI). For categorical variables, n (%) is reported and, for continuous variables, the mean (SD)/median (min;max). For comparisons between ordered groups, the Mantel–Haenszel chi square test was used for dichotomous and ordered categorical variables, while the Jonckheere-Terpstra test was used for continuous variables. Univariable and multivariable logistic regression were used for predictors of SSIs, unadjusted and adjusted for age and gender. A multivariable logistic regression analysis was performed. The variables were included together and selected, based on the results of the univariable analysis, and, for clinical relevance, years 1–5, gender, procedure time, sepsis and skin damage. “Urinary tract catheterization (UTC) more than once” was not included, because the association with SSI is multifactorial. It is probable that an indwelling urinary catheter (IUC) can be the result of an SSI rather than a cause.

*P*-values, odds ratios (OR) with a 95% confidence interval and area under the ROC curve are based on original values and not on stratified groups. All significance tests were two-sided and conducted at the 5% significance level. Data were analysed with the SPSS statistical package version 25 (IBM Corp. Armonk, NY, USA) and SAS Version 9.4, SAS Institute, Cary, NC, USA.

## Results

The results are based on an analysis of data comprising 3553 patients. Patient and clinical characteristics are presented in Table 4. There were few changes in the case mix over the years, although there were more patients with cognitive impairments in years 4–5 compared with years 1–2. HLOS decreased by three days from years 1–2 to years 4–5.

**Table 4:** Patient characteristics and clinical data over five years

Variable	1 (n = 442) Baseline	2 (n = 820) Intervention	3 (n = 806) Intervention	4 (n = 773) Follow-up	5 (n = 712) Follow-up	*p*-value
*Patient characteristics*						
Women (n, %)	312 (70.6%)	547 (66.7%)	551 (68.4%)	540 (69.9%)	490 (68.8%)	0.77
Age (mean, SD)	83.5 (8.2) 84.5 (65; 101) n = 442	84.5 (7.8) 85 (65; 102) n = 820	83.8 (8.2) 85 (65; 101) n = 806	83.8 (8.3) 85 (65; 102) n = 773	83.6 (8.3) 84 (65; 104) n = 712	0.19
ASA classification						
I	20 (4.5%)	15 (1.8%)	14 (1.7%)	30 (3.9%)	17 (2.4%)	
II	180 (40.7%)	334 (40.8%)	289 (35.9%)	315 (40.9%)	286 (40.5%)	
III	211 (47.7%)	410 (50.1%)	448 (55.6%)	382 (49.6%)	355 (50.2%)	
IV	31 (7.0%)	60 (7.3%)	55 (6.8%)	43 (5.6%)	49 (6.9%)	0.88
Diabetes	61 (13.8%)	126 (15.4%)	128 (15.9%)	133 (17.2%)	123 (17.3%)	0.072
Cognitive failure	125 (28.3%)	267 (32.6%)	352 (43.7%)	315 (40.8%)	258 (36.2%)	0.0005
Housing						
Home	370 (83.7%)	573 (69.9%)	568 (70.5%)	516 (66.8%)	504 (70.8%)	
Nursing home	72 (16.3%)	247 (30.1%)	238 (29.5%)	257 (33.2%)	208 (29.2%)	< .0001
Intact skin on admission	291 (66.6%)	525 (64.9%)	496 (62.2%)	514 (66.9%)	457 (64.2%)	0.81
*Clinical data*						
Time to surgery from admission (hours)						
< 36	399 (90.3%)	702 (85.7%)	641 (79.5%)	627 (81.1%)	626 (88.0%)	
> 36	43 (9.7%)	117 (14.3%)	165 (20.5%)	146 (18.9%)	85 (12.0%)	0.21
Length of surgery	75.1 (34.3) 72 (9; 226) n = 442	73.7 (32.4) 70 (13; 208) n = 820	70.2 (30.4) 69 (9; 248) n = 805	75.8 (34.3) 74 (11; 222) n = 773	71.5 (30.0) 69.5 (13; 174) n = 712	0.71
Length of hospital stay	14.3 (7.3) 13 (3; 60) n = 442	13.6 (7.6) 13 (2; 68) n = 820	12.4 (7.7) 11 (2; 133) n = 806	10.7 (5.4) 10 (3; 46) n = 772	9.7 (4.76) 9 (2; 44) n = 712	< .0001
Surgical method						
Total arthroplasty	44 (10.0%)	75 (9.1%)	88 (11.1%)	83 (10.8%)	65 (9.1%)	
Hemiarthroplasty	134 (30.3%)	274 (33.4%)	247 (31.1%)	244 (31.9%)	218 (30.6%)	
Internal fixation	264 (59.7%)	470 (57.3%)	459 (57.8%)	439 (57.3%)	426 (59.8%)	0.74
Missing	0 (0.0%)	1 (0.1%)	0 (0.0%)	0 (0.0%)	3 (0.4%)	
In-dwelling UTC^a^ more than once	24 (10.7%)	83 (15.7%)	101 (18.2%)	76 (11.2%)	82 (12.8%)	0.22

For categorical variables, n (%) is presented

For continuous variables, the mean (SD)/median (range)/n = is presented

For comparisons between groups, the Mantel–Haenszel chi square test was used for ordered categorical variables and the Jonckheere-Terpstra test was used for continuous variables

^a^In-dwelling Urinary Catheter (UTC)

### Surgical site infections

The frequency of early SSIs decreased from 2.5% in years 1–2 to 1.1% in years 4–5 (Table 4). The overall frequency SSI in hemiarthroplasty was 2.7%, while it was 1.5% for internal fixation.

In the univariable analyses (adjusted for age and gender), an earlier year in the study period, gender, sepsis, severe pressure ulcer (Norton stage 4), skin damage, sepsis and “UTC more than once” significantly increased the odds of developing an SSI (Table 5).

**Table 5:** Univariable predictions of SSI adjusted by age and gender

Variable	1 (n = 442) Baseline	Year 2 (n = 820) Intervention	Year 3 (n = 806) Intervention	Year 4 (n = 773) Follow-up	Year 5 (n = 712) Follow-up	*p*-value
SSI^a^	13 (2.9%)	18 (2.2%)	19 (2.4%)	9 (1.2%)	8 (1.1%)	0.0079
UTI^b^	75 (17.0%)	104 (12.7%)	58 (7.2%)	31 (4.0%)	31 (4.4%)	< .0001
Sepsis	13 (2.9%)	21 (2.6%)	23 (2.9%)	12 (1.6%)	8 (1.1%)	0.0094
Pneumonia	35 (7.9%)	71 (8.7%)	48 (6.0%)	45 (5.8%)	63 (8.9%)	0.76
Covid-19	0 (0.0%)	0 (0.0%)	0 (0.0%)	0 (0.0%)	12 (1.7%)	
Any type of infection except SSI	124 (28.1%)	216 (26.3%)	163 (20.2%)	111 (14.4%)	131 (18.4%)	< .0001

For categorical variables, n (%) is presented

For comparisons between groups, the Mantel–Haenszel chi square test was used for ordered categorical variables

^a^Surgical site infections

^b^Urinary tract infection requiring medical treatment

Patients with SSIs had twice as long a mean HLOS of 22.5 days (95% CI 18.0–27.1) compared with 11.8 days without SSIs (95% CI 9.1–12.49) *p* < 0.0001. Patients with early SSIs also suffered from more other infections than those without: UTI 13.3% versus 8.3%, sepsis 11.9% vs 4.7% and pneumonia 10.5% vs 7.0%.

The multivariable regression model results suggest that (OR: 95% CI); year (0.77: 0.64–0.94), male gender (1.71:1.03–2.82), procedure time (the OR displays changes per 1 min) (1.01: 1.0–1.02), sepsis (4.58: 1.98–10.59) and skin damage (1.67: 1.01–2.75) contributed significantly to the model, the area under the ROC curve, with (95% CI) = (0.69:0.63–0.79).

### Hospital-acquired infections

Significant reductions in HAIs other than SSIs were also seen for UTI (14.2% to 4.2%) and sepsis (2.7% to 1.3%). No significant differences were observed for pneumonia (Table 6).

**Table 6:** Infectious outcomes over five years

		Univariable*			Adjusted**		
Variable and values	n (%) of events	OR (95%CI)	*p*-value	Area under ROC curve (95%CI)	OR (95%CI)	*p*-value	m^a^
*Year 1–5*							
1	13 (2.9%)						
2	18 (2.2%)						
3	19 (2.4%)						
4	9 (1.2%)						
5	8 (1.1%)	0.78 (0.64–0.94)	0.0086	0.59 (0.53–0.66)	0.78 (0.64–0.94)	0.0092	
*Gender*							
Female	36 (1.5%)						0
Male	31 (2.8%)	1.91 (1.18–3.11)	0.0088	0.58 (0.52–0.64)	1.97 (1.21–3.22)	0.0066	
*Age*							
65– < 81	22 (1.9%)						0
81– < 89	22 (1.7%)						
89–104	23 (2.0%)	1.01 (0.98–1.04)	0.57	0.52 (0.45–0.59)	1.01 (0.98–1.05)	0.37	
*ASA*							
I	0 (0.0%)						9
II	19 (1.4%)						
III	43 (2.4%)						
IV	5 (2.1%)	1.52 (1.05–2.21)	0.027	0.57 (0.52–0.63)	1.41 (0.96–2.06)	0.078	
*Diabetes*							
Yes	12 (2.1%)						0
No	55 (1.8%)	0.88 (0.47–1.65)	0.68	0.51 (0.46–0.56)	0.93 (0.49–1.75)	0.81	
*Hospital length of stay (days)*							
2- < 9	8 (0.7%)						
9- < 14	10( 0.8%)						
14–133	49 (4.3%)	1.11 (1.09–1.13)	< 0.0001	0.76 (0.70–0.82)	1.11 (1.08–1.13)	< 0.0001	1
*Cognitive failure*							
Yes	25 (1.9%)						1
No	42 (1.9%)	0.99 (0.60–1.63)	0.97	0.50 (0.44–0.56)	1.05 (0.63–1.75)	0.84	
*Time to surgery*							
< 36 h	51 (1.7%)						2
> 36 h	16 (2.9%)	1.71 (0.97–3.02)	0.065	0.54 (0.49–0.59)	1.72 (0.97–3.05)	0.064	
*Procedure time, minutes*							
9– < 57	17 (1.4%)						1
57– < 85	14 (1.2%)						
85–248	35 (2.9%)	1.29 (1.05–1.59)	0.013	0.59 (0.51–0.66)	1.31 (1.07–1.62)	0.0095	
*Pressure ulcers, Norton scale 1–4*							
1 versus no	8 (4.4%)	2.10 (0.95–4.63)	0.066		2.06 (0.93–4.56)	0.076	1732^b^
2 versus no	2 (1.8%)	0.83 (0.20–3.50)	0.80		0.82 (0.19–3.48)	0.79	
3 versus no	1 (6.7%)	3.28 (0.42–25.71)	0.26		3.37 (0.43–26.59)	0.25	
4 versus no	2 (25.0%)	15.31 (2.98–78.79)	0.0011	0.57 (0.50–0.64)	13.26 (2.53–69.35)	0.0022	
Urinary tract infection	9 (3.0%)	1.71 (0.84–3.49)	0.14	0.53 (0.48–0.57)	1.68 (0.82–3.43)	0.15	
Sepsis 0	59 (1.7%)						1
Any Sepsis	8 (10.4%)	6.71 (3.09–14.58)	< 0.0001	0.55 (0.51–0.59)	5.98 (2.73–13.10)	< 0.0001	
Pneumonia	8 (3.1%)	1.72 (0.82–3.65)	0.15	0.52 (0.48–0.56)	1.55 (0.73–3.30)	0.25	
Any type of infection except SSI	21 (2.8%)	1.74 (1.03–2.94)	0.037	0.55 (0.50–0.61)	1.63 (0.97–2.76)	0.068	
Skin damage	32 (2.6%)	1.81 (1.11–2.95)	0.018	0.57 (0.51–0.63)	1.66 (1.01–2.73)	0.046	30
Before and after one CHX^c^ shower was implemented	27 (2.3%)						1
	39 (1.6%)	0.71 (0.43–1.17)	0.18	0.54 (0.48–0.60)	0.72 (0.44–1.18)	0.19	
IUC^d^ more than once	18 (4.9%)	3.60 (2.00–6.49)	< 0.0001	0.61 (0.54–0.68)	3.30 (1.81–6.01)	< 0.0001	

P-values, OR and area under ROC curve are based on original values and not on stratified groups

OR is the ratio for the odds of an increase in the predictor of one unit. For procedure time, the odds is given per 30 min

M^a^ = missing data

^b^ = pressure ulcers, Norton stage 1–4, recorder years 1–3

CXH^c^ = chlorhexidine gluconate

^d^ = in-dwelling catheterisation more than once during hospital stay

*All tests are performed with univariable logistic regression

**Adjusting for gender and age using logistic regression

Of 77 patients with sepsis in this cohort, 20% were diagnosed on admission and 80% were hospital associated. Fifty-nine % were of unknown origin, 14% and 5% secondary to a UTI and pneumonia respectively. The mean HLOS for patients without sepsis was 12 days (95% CI 11.6–12.1), while it was 23 days for those with hospital-associated sepsis (95% CI 15.6–31.1).

## Discussion

During the study period, we observed that in-hospital SSIs and other nosocomial infections following treatment and care for hip fractures can be significantly reduced by using the bundle approach based on the *Safe Hands* project. The initial early SSI rate in our cohort lies in the mid-range of previously reported rates [3] and the rate after implementation of the bundle interventions was in the lower range [23]. Rates of pneumonia and unspecified infection increased slightly in the fifth study year, and this is probably attributable to Covid-19. For every year, the odds of an early SSI decreased, despite that there were significantly more patients with cognitive impairment and nursing home residents in the last two study years, indicating greater frailty in the cohort [24]. In line with the literature, male gender, prolonged procedure time and more than one urinary catheterisation increased the odds of early SSIs [2, 3, 16]. In contrast, age, diabetes, and an ASA score did not predict SSI in our cohort. The identification of discrete modifiable risk factors is clinically desirable to ensure optimal intervention. The strong association between sepsis and SSI was not surprising [25]; it stresses the importance of handling all medical devices, such as venous and urinary catheters, with strict adherence to hand hygiene guidelines and aseptic techniques [26]. *S. aureus* bacteremia, albeit to a lesser extent if hospital acquired, increases the risk of bacterial seeding to a previously inserted orthopaedic implant or another biomedical device, thereby compounding morbidity [27–29]. There are studies from several centres including our hospital that report decreased mortality and hospital re-admission in key infections, including *S. aureus* bacteremia, following early Infectious disease consultations [30, 31]. To this end, *S. aureus* alarms and the increased availability of bedside ID consultations were introduced in the second quarter of the first study year and in the first quarter of the third study year, respectively. Skin lesions on admission and severe pressure ulcers constituted a very high risk of SSI. The latter is a modifiable risk factor that shows the importance of a team effort preventing complications in surgery; by including RNs and emphasising the importance of optimal nursing care, pressure ulcers can be avoided. However, there is a need for high-quality trials, establishing the optimal repositioning frequency in this patient group [32].

### Strengths and limitations

As this is a single-centre observational study, there are caveats when it comes to the interpretation of these results. In addition, other changes in hospital standard practices, many of which were developed in relation to the growing organisational focus on this patient group, have probably impacted the results of the *Safe Hands* interventions. What we can see is a probable reciprocal effect where the different changes reinforce the results in terms of patient outcome. However, we have transparently reported all the changes that have occurred during the five years included here to minimise the risk of overstating the influence the *Safe Hand*s project has had on clinical practice. The study’s strengths include the large study cohort of 3553 patients and longitudinal nature. To avoid imputation errors, the registered data have been validated against patient records. The local quality register started in 2015 and the number of patients included in it has fluctuated over the years. Fewer patients were included in the first years of the register. The estimated completeness in the first year was approximately 60%, based on a median value of included patients in years 2 to 5. No systematic errors that can explain the lack of imputation in the first year of the register have been found.

Using only routinely collected data to analyse outcome has its limitations. As a result, other important prognostic and confounding factors, such as blood transfusion, body weight and smoking, have not been controlled for in the statistical analysis.

Bundle approaches have inherent strengths and limitations. Previous bundle interventions have proven useful in improving the quality of care and reducing SSIs in HF patients [6, 23] and other serious HAIs, such as blood-stream infections [33] and ventilator-associated pneumonia [34]. Others have criticised bundle approaches and challenged their usefulness, as it is difficult or even impossible to tease out the parts of the bundle that have contributed to the desired change and the extent. We argue that this criticism is less important than the potential benefits of bundles. Moreover, it might be useful to move away from linear thinking where every single part can be measured and understood, to acknowledge the complexity of change and view the transformation process from a holistic perspective where the whole is greater than the simple sum of parts.

### Lessons learned

At the start of the *Safe Hands* project, we aimed to create sustained improvements in the treatment and care of older individuals with hip fracture, with special emphasis on infection prevention. The results of the present study indicate sustained improvements and, moreover, the incidence of early SSIs, UTIs and bloodstream infections continued to decrease even after the interventions were implemented in year three and the research team left the site. It is common for most interventions to show an effect over the short term, but the challenge has been to create sustained improvements after the intervention [35], a challenge we were aware of when deciding on the implementation strategy. We see some explanations of our promising results and sustained effect. Implementation theories and frameworks have highlighted how contextual factors can both promote and hinder the uptake of evidence-based care [36–39]. For this reason, the results of our study cannot be understood without acknowledging contextual mechanisms such as leadership engagement, resources, an organisational safety culture and commitment to change. To add another layer of complexity, the *Safe Hands* implementation programme was aimed at surgeons, RNs, specialised RNs and nurse assistants, leaders (formal and informal) and managers. To handle this complexity, the programme was based on facilitating mechanisms for contextual negotiation and collective action; (1) Building a strong *partnership* between researchers, management and clinicians based on mutual respect, (2) *External and internal facilitation* as a role and a process that focused on enabling and supporting individual and organisational learning [19, 40]. We found that the choice of facilitators was critical and needed to be adapted to match the context. To be perceived as trustworthy, these facilitators needed to have an in-depth understanding of the medical context and infection prevention. The internal facilitators were introduced in a staged manner and represented all the professional categories. When the external facilitator left the site, the internal facilitators remained and were able to function as local champions [15]. In this way, the improvements and learning in clinical practice could continue and may be one contributory factor in terms of the sustained and reduced infection rates. (3) *Dialogue* and *co-creation,* to facilitate organisational learning. Isaacs’ [18] and Schein’s [17, 41, 42] work has demonstrated the significance of creating space for dialogue. From their work, we used interprofessional dialogue to learn more about one’s own and co-workers’ ways of thinking about infection prevention and to inquire collectively about how available knowledge could best be transformed into co-creating and testing new ways of working together to reduce the risks of infection after surgery. As a result, the work aimed to create a cultural change instead of modifying behaviours. For this to occur, we found, in line with previous studies [17, 43] (p. 305), that the creation of psychological safety, mediated by respectful dialogue, was imperative to facilitate transformation.

Initially, very few people in the organisation appeared to acknowledge the magnitude of the problem with HAIs. Competing interests and other daily problems to resolve may have shadowed the infection issue. By using local quality data as a basis for dialogue with the management and clinicians lead to increasing awareness and a shared sense of urgency in relation to the problem. Most managers and clinicians developed the motivation to engage in the transformative work, even if not everyone was motivated to make changes. To sum up, the *Safe Hands* project changed the way risks, safety and infection prevention were perceived in relation to hip fracture patients [15] and significantly improved patient outcomes.

## Conclusions

Our preventive bundle, based on partnership between researchers, managers and clinicians and a strong commitment to change from the involved professions, appear to be effective in reducing the frequency of potentially devastating SSIs and other HAIs after hip fractures. The use of external and internal facilitators was crucial to enable individual and organisational learning and overcoming barriers to improvements.

## Data Availability

Data are available in response to reasonable requests.
